# Skewed X-chromosome inactivation in patients with esophageal carcinoma

**DOI:** 10.1186/1746-1596-8-55

**Published:** 2013-04-04

**Authors:** Gang Li, Tianbo Jin, Hongjuan Liang, Yanyang Tu, Wei Zhang, Li Gong, Qin Su, Guodong Gao

**Affiliations:** 1 Department of Neurosurgery, Tangdu hospital, the Fourth Military Medical University, Xi’an, 710038, China; 2 National Engineering Research Center for Miniaturized Detection Systems, School of Life Sciences, Northwest University, Xi’an, 710069, China; 3 Department of Clinical Experimental Surgery, Tangdu hospital, the Fourth Military Medical University, Xi’an, 710038, China; 4 Department of Pathology, Tangdu Hospital, the Fourth Military Medical University, Xi’ an, 710038, China; 5 Department of Pathology, Cancer Hospital, Chinese Academy of Medical Sciences and Peking Union Medical College, Beijing, 100021, China

**Keywords:** Skewed X-chromosome inactivation, Androgen receptor gene, Carcinoma, Esophagus, Cancer predisposition

## Abstract

**Abstract:**

Skewed X-chromosome inactivation (SXCI) was found in some apparently healthy females mainly from Western countries. It has been linked to development of ovarian, breast and pulmonary carcinomas. The present study aimed to observe the SXCI frequencies in apparently healthy Chinese females and patients with esophageal carcinoma. DNA was extracted from the peripheral blood cells from 401 Chinese females without a detectable tumor and 143 female patients with esophageal carcinoma. Exon 1 of androgen receptor (*AR*) gene was amplified, and the products of different CAG alleles were resolved on denaturing polyacrylamide gels and visualized after silver staining. The corrected ratios (CR) of the products before and after *Hpa*II digestion were calculated. As to the healthy females, when CR ≥ 3 was used as a criterion, SXCI was found in two (4.3%) of the 46 neonates, 13 (7.8%) of the 166 younger adults (16–50 years) and 37 (25.7%) of the 144 elderly females (51–96 years), with the frequency higher in the elderly subjects than in the two former groups (*P* < 0.05). When a more stringent criterion (CR ≥ 10) was used, SXCI was found in one (2.2%), two (1.2%) and 16 (11.1%) of the subjects in the three age groups, respectively, itsfrequency being higher in the elderly than in the younger age groups (*P* < 0.05). Occurrence of SXCI was detected in both the patients and controls at similar frequencies. However, the phenomenon, as defined as CR ≥ 3, was more frequent in the patients aging <40 years (35.7%) compared to the corresponding reference group (7.6%, *P* = 0.006). When CR ≥ 10 was adopted, the frequencies were 7.1% and 1.2%, respectively. Their difference did not attain statistical significance (*P* = 0. 217). SXCI also occurs in apparently healthy Chinese females, and is associated with age. It may be considered as a predisposing factor for the early development of esophageal carcinoma.

**Virtual slides:**

The virtual slide(s) for this article can be found here http://www.diagnosticpathology.diagnomx.eu/vs/1542364337927656

## Introduction

In female mammals one of the two X chromosome is inactivated in early embryonic life. Females are therefore mosaics for two cell types, cells with the maternal X chromosome (Xm) as the active one, and cells with the paternal X chromosome (Xp) as the active X one [1,2]. Theoretically, the ratio of the inactive paternal X-linked allele to the inactive maternal one should be 1:1, and any significant deviation from the ratio is termed as skewed X-chromosome inactivation (SXCI) [3,4]. The Lyonization ratio (inactive Xp/inactive Xm) of a large population of females follows a Gaussian distribution pattern in which SXCI is a statistically rare event [5].

SXCI was incidentally observed when Vogelstein and his colleagues were establishing a clonality assay based on the human androgen receptor (*AR*) gene polymerase-chain reaction (PCR) [3]. The phenomenon has been observed and SXCI frequencies varied from 17% to 65% in apparently healthy females as revealed by several surveys from Western countries [5-12]. While its incidence was found to increase with age [5-12], some groups failed to prove the association [13,14]. Yet, up to date little is known about whether there is such an association in apparently healthy Chinese females or not.

Though SXCI has been associated to development of breast [15-17], ovarian [18] and pulmonary carcinomas [19], however, the clinical significance of SXCI are still to be elucidated. Does the imbalanced inactivation of X chromosomes in female somatic cells, as usually demonstrated using peripheral blood sample, indicate an increased risk of malignant solid tumours development? To our knowledge, esophageal carcinoma (EC) ranks as the tenth most prevalent malignant solid tumours in the world, with marked regional variation and a particularly high incidence in certain regions of China [20-22]. Albeit current evidence suggests that inherited risks play a significant role in esophageal carcinoma susceptibility, and several molecular alterations have been found in esophageal carcinoma [20,22], the underlying pathomechanisms remain elusive. Thus, the present study described the frequency of SXCI in apparently healthy Chinese females and linked the phenomenon to early development of esophageal carcinoma.

## Subjects and methods

### Subjects and DNA extraction

Peripheral blood was taken from 143 female patients with esophageal carcinoma and 401 apparently healthy females. The patients were admitted in Tangdu hospital, Fourth Medical Military University in Xi’ an, China during the period from April 2003 to October 2010, with all of the cases diagnosed as a primary esophageal carcinoma through endoscopy and/or surgical resection of the tumour, and pathological examination. The patients were 35 to 83 (median, 61) years old at diagnosis, without a record of smoking or a family history of esophageal carcinoma. Among them, 141 patients had squamous cell carcinoma, and 2 had adenosquamous carcinoma. Clinical stages were evaluated according to the AJCC staging system [23], with 92, 13, 10, 16, 12 cases determined as stages I, IIA, IIB, III and IV, respectively. A total of 401 apparently healthy females were recruited from April 2003 to September 2010. They were from 2 days to 96 (median, 62) years of age, without a smoking history or any personal or family record of a genetic or a neoplastic disease. Among them, 69 were staff members of Tangdu Hospital who received regular check-ups, 52 were healthy neonates (below 4 weeks of age) born in the Department of Obstetrics, Tangdu Hospital, and the remaining 280 were female patients admitted to the Department of General Surgery of Tangdu Hospital for surgical reasons other than a tumor or genetic diseases. It was stated that 289 females of the 401 healthy ones, were previously assessed and reported on [19]. A written informed consent was obtained from all the subjects or their custodians, and we collected all the blood samples from the patients before chemotherapy or radiotherapy. The protocol was approved by the Medical Ethics Committee of the Fourth Military Medical University.

Peripheral blood was taken from the elbow vein or the head superficial vein, and treated immediately with an anticoagulant containing sodium citrate (22 g/L) and sodium chloride (8.5 g/L). The samples were then stored at −70°C before use. Genomic DNA was isolated from the samples by using an extraction kit (Qiagene, Hilden, Germany). DNA concentration and purity were determined by an ultraviolet spectrophotometer (Eppendorf, Hamburg, Germany).

### Principles of X-chromosome inactivation analysis

The analysis is based on differential inactivation of X chromosomes of female somatic tissues and the CAG short-tandem repeat (STR) polymorphism at the *AR* gene exon 1 [24]. There are two *Hha*I and two *Hpa*II restriction sites at the locus 100 bp upstream to the CAG STR with a heterozygosity frequency of around 90% [24,25]. X-chromosome inactivation is associated with the methylation of these restriction sites. When these sites are methylated, as on the inactive X chromosome, the gene can not be transcribed, whereas when unmethylated, as on the active X chromosome in females or on the male X chromosome, the gene can be transcribed [18]. The digestion with methylation-sensitive endonucleases, followed by PCR with primers flanking these restriction sites and the highly polymorphic STR, can be used to distinguish between the transcriptionally active and inactive X chromosome in heterozygous female subjects.

In females with random X-chromosome inactivation, the amplification products from both alleles should be equal, with a ratio of approximately 1:1. In the neoplastic tissues most of which originate from single cell clones [19], the ratio changes markedly compared with the surrounding normal tissues. The nonrandom X-chromosome inactivation has been used in the description of clonality status of lesions with undetermined nature [26,27]. Meanwhile, a remarkable deviation of the ratio has been observed in apparently non-neoplastic cell populations, such as peripheral blood cells of some females, which is defined as SXCI [3,4].

### Analysis of skewed X chromosome inactivation (SXCI)

DNA was digested by mixing 10 μL (1 μg) of sample DNA with 0.5 μL of *Hpa*II (10 U/μL; Promega, Madison, WI, USA), 2 μL of 10 mol/L reaction buffer, 0.2 μL of 10 g/L bovine serum albumin and 7.3 μL of deionized water. The mixture was then incubated at 37°C for 4 h and the reaction was terminated by incubation at room temperature for 30 min as suggested by the manufacturer. Nested PCR was conducted as described previously [19]. A negative, water-blank control was always included in each batch of PCR. If the negative control was shown to be positive, the reaction was repeated for the whole batch. The reaction fidelity of *Hpa*II digestion was guaranteed by parallel negative controls with the enzyme omitted from the reaction mixture. In addition, the whole assay was carried out twice in independent series.

Amplification efficacy was demonstrated through electrophoresis on 2% agarose gels. The amplification products with 4 μL for each were mixed with the same volume of loading buffer (1 g/L xylene cyanole, 1 g/L bromophenol blue, in formamide), loaded onto the 10% polyacrylamide gel containing 8 mol/L urea, resolved through electrophoresis with the Mini-VE system (Amersham Biosciences Corp., San Francisco, CA) at a voltage of 80 v for 8 h, and then visualized after silver staining as described previously [25]. For the samples whose allelic differences at the CAG STR were small (one or two repeats), a longer gel (26-cm long and 0.75-mm thick) was used for the resolution with the SE660 system (Amersham). The results were recorded, and the intensities of the products from both alleles were analyzed by using an image-analyzing system (LabWorks 3.0, UVP, Cambridge, UK).

In order to avoid the interference of possible preferential amplification of one of the alleles, we used the corrected ratio (CR) to evaluate the X-chromosome inactivation pattern by comparing the allelic difference of a sample before and after HpaII digestion. CR was derived by dividing the ratio of the upper-band intensity to the lower-band intensity of the sample after digestion by that of the same sample before digestion. If CR was <1, the reciprocal value was considered. In the present study, CR ≥ 3, which indicated the expression of the same allele in above 75% of the cells examined, was used to define SXCI. In addition, we also used CR ≥ 10 as a more stringent criterion for defining SXCI.

### Statistical analysis

Statistical analysis was performed using an SPSS package (Version 13.0; SPSS Inc., Chicago, Illinois) for Windows. The likelihood ratio test was performed to determine the difference SXCI frequency among various age groups. The χ^2^ test was also used for comparison of categorical variables. A *P* value of <0.05 (two-tailed) was considered statistically significant.

## Results

**Figure 1:**
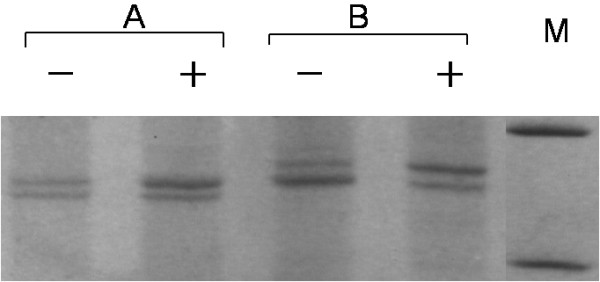
**Representative data of *AR *gene exon 1 amplification products of two subjects (A and B) with CAG STR polymorphism.** +, with *Hpa*II digestion; -, without *Hpa*II digestion; M, DNA marker, the upper and lower bands representing positions of 300 and 200 bp, respectively.

Amplification for AR gene exon 1 was successful in all samples from the subjects. Among the 401 healthy females, 356 (88.8%) were shown to be polymorphic at the CAG STR (Figure 1), and hence informative for X-chromosome inactivation analysis. The ages of the informative cases ranged from 2 days to 96 years, with a median age of 63 years.

**Figure 2:**
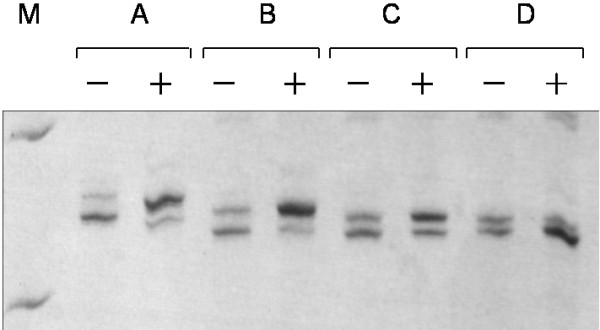
**Representative data of X-chromosomal inactivation patterns in subjects, with SXCI in samples from subjects A (CR = 14, >10), B (CR = 19, >10) and C (CR = 4, >3) and without SXCI in the sample from subject D (CR = 2.1, <3).** M, DNA marker, with the upper and lower bands at positions of 300 and 200 bp, respectively.

**Figure 3:**
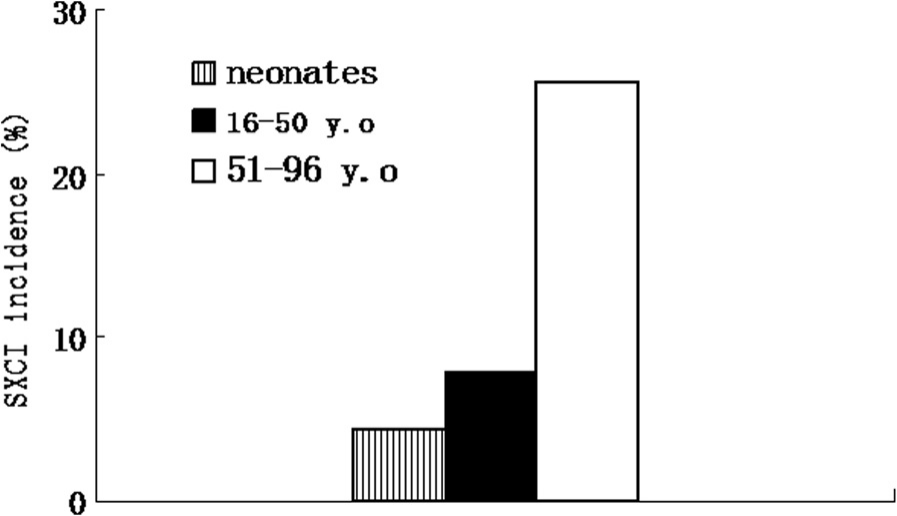
Incidence of SXCI (CR ≥ 3) in normal females from three age groups.

Both CR ≥ 3 and CR ≥ 10 were used as criteria for SXCI to describe the X-chromosomal inactivation skewing in the subjects (Figure 2). Among them, 52 (14.6%) were found to have a CR value more than or equal to 3 and 19 (5.3%) had a CR value more than or equal to 10. Frequencies of SXCI (CR ≥ 3) in various age goups were determined, being 4.3% (2/46), 4.9% (3/61), 9.5% (10/105), 10.8% (7/65) and 38.0% (30/79), respectively, in the neonates and those who aged 16 to 30, 31 to 50, 51 to 70 and 71 to 96 years. For age-matched comparision to the patient groups as described below, the 356 informative healthy subjects were further divided into three age groups including neonates (2 to 28 days of age; median, 19days; *n* = 46), younger adults (16 to 50 years of age; median, 41 years; *n* = 166) and the elderly subjects (51–96 years of age; median, 73 years; *n* = 144). When CR ≥ 3 was taken as the criterion, SXCI frequencies were 4.3% (2/46), 7.8% (13/166) and 25.7% (37/144), respectively, in the neonate, younger adult and elderly groups. When CR ≥ 10 was used as the criterion, SXCI was found in only one neonate (2.2%), two (1.2%) younger adults and 16 (11.1%) elderly females. SXCI frequency was significantly associated with age (*P <* 0.001), and the frequency in the elderly was higher than that in younger adults (*P* < 0.05) or in the neonates (*P* < 0.05) whether CR ≥ 3 or CR ≥ 10 was used as a criterion. SXCI appeared to be more frequent in the adults than in neonates, when CR ≥ 3 was used as the criterion (4.3% and 16.1%, *P* = 0.041) (Figure 3), while the difference between neonates and the younger adults without a detectable cancer did not attain statistic significance, whether using CR ≥ 3 or CR ≥ 10 as the criterion.

Among the 143 patients with esophageal carcinoma, 134 were shown to be polymorphic at the CAG STR, and thereby informative for X-chromosomal inactivation analysis. Their ages at diagnosis ranged from 35 to 83 years, with their median being 63 years. No significant difference was found between the polymorphism frequencies for the cancer patient and control groups (93.7% *vs* 88.8%, *P* > 0.05). With these data combined, the frequency of CAG STR polymorphism was 90.1% (490/544), being similar to that from other authors [5,7,12,13,15,18,19,28-32].

SXCI frequeny was evaluated for patients with esophageal carcinoma, and compared to that of the referenc group (Table 1). When CR ≥ 3 was adopted to determine SXCI, the frequencies were 9.7% and 8.8%, respectively. When CR ≥ 10 was adopted, the frequencies were 5.2% and 2.8%, respectively. The differences were not significant (*P* > 0.05). However, the frequency in the patients aging ≤40 years (35.7%) was found to be higher than that in the corresponding references without a detectable cancer (7.6%) as SXCI defined as CR ≥ 3 (*P* = 0.006). When CR ≥ 10 adopted, the frequencies were 7.1% and 1.2%, respectively, and their difference did not attain statistical significance (*P* = 0. 217). In the patients and references beyond 40 years, the excessive skewing, as defined by CR ≥ 3 and CR ≥ 10, were observed in 6.7%, 10.4% and 5.0%, 5.2% of the subjects, respectively. No significant difference was found between these two groups (*P* > 0.05).

**Table 1:** Skewed X-chromosomal inactivation frequencies in esophageal carcinoma patients of various age ranges and the corresponding controls

**Groups**	**Age (years)**	**Numbers examined**	**Numbers with CR ≥ 3(%)**	***P-*value**	**Numbers with CR ≥ 10(%)**	***P-*value**
Patients	≤40	14	5 (35.7)	0.006	1 (7.1)	0.217
Controls		170	13 (7.6)		2 (1.2)	
Patients	>40	120	8 (6.7)	0.301	6 (5.0)	0.940
Controls		115	12 (10.4)		6 (5.2)	
Patients	35-83	134	13 (9.7)	0.757	7 (5.2)	0.214
Controls	16-83	285	25 (8.8)		8 (2.8)	

**Figure 4:**
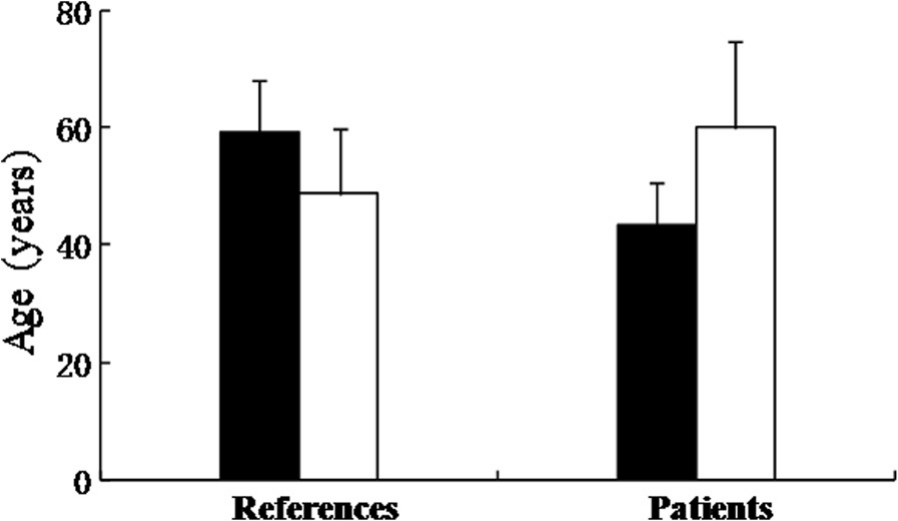
**Average ages of the esophageal carcinoma patients and references with SXCI defined by CR ≥ 3 (filled bar) and without SXCI (empty bar).** Their standard deviations denoted on each bars. The differences in both the control and patient groups are statistically significant, with their *P* values below 0.05 and 0.005, respectively.

The average ages of the subjects with and without SXCI were determined for both groups (Figure 4). Whether CR ≥ 3 or CR ≥ 10 was taken as the criterion, average age of the apparently healthy female sujects with SXCI was more than 10 years older than that of those without SXCI (*P* < 0.05). In contrast, average age of the cancer patients with SXCI was more than 16 years younger than that of those without SXCI (*P* < 0.01; Figure 4).

The relationship between SXCI and clinical stages of the cancer was assessed. With the criterion of CR ≥ 3 adopted, the frequencies for the cases of stages I, IIA, IIB, III and IV were 8.0% (7/88), 20.0% (2/10), 10.0% (1/10), 13.3% (2/15), and 9.1% (1/11), respectively. There was no significant difference among them (*P* > 0.05).

## Discussion

SXCI was described in 1987 during a clonality assay based on *AR* gene polymorphism using tissue samples [3]. After then, the phenomenon was also observed in peripheral blood cells in the surveys of X chromosomal inactivation patterns in apparently healthy American females by Gale *et al.*[4] and Busque *et al.*[5]. Its occurrence was associated with age, with SXCI (CR ≥ 3) frequencies being estimated to be 8.6%, 16.4% and 38.0%, respectively, in neonates, young (28 to 32 years) and elderly (≥60 years) female adults [5]. Data from several other groups confirmed the SXCI association with age [5-12,32], while some authors failed to approve it [13,14]. In the present study, SXCI was also observed in peripheral blood cells from apparently healthy Chinese females without a detectable tumor. The SXCI (defined by CR ≥ 3) frequencies were estimated to be 4.3%, 7.8% and 25.7%, respectively, in the neonates, younger adults (16 to 50 years) and the elderly (beyond 50 years) (Table 2). The present study shows that SXCI also occurs in neonates, albeit infrequently, and provide furthur evidence for the link of SXCI to ageing.

**Table 2:** SXCI (CR ≥ 3) incidences in healthy females of various age ranges and ethnic groups

**Authors**	**Subjects**	**Numbers examined**	**Age range (years)**	**Overall Incidences (%)**	**SXCI (CR ≥ 3) incidences of healthy females of various age ranges (%)**								***P-*value**
					**0**	**25- 32**	**16- 50**	**35- 50**	**51- 70**	**≥60**	**≥75**	**71- 96**	
Lau *et al.*[8]	Canadian	27	35-50	33	*	*	*	33	*	*	*	*	*
Busque *et al.*[5]	American	295	0-96^☆☆^	17	9	16	*	*	*	38	*	*	<0.0001**
Gale *et al.*[6]	English	174	17-96	38	*	*	22	*	*	*	6	*	< 0.0001*
Tonon *et al.*[7]	Italian	68	25-32, ≥75	23	*	17	*	*	*	*	45	*	< 0.02^☆^
Knudsen *et al.*[11]	Norwegian	80	19-90	65	*								0.034**
Mossner *et al.*[12]	German with Caucasian background	139	0-40, 41–64, 65-97	34^§^	*								0.0008**
Racchi *et al.*[13]	Italian	166	8-94	30	*	23	26	*	*	37	40	*	>0.05**
Gentilini *et al.*[28]	Italian	148	31-100	38.5	*								<0.0001**
Amos-Landgraf *et al.*[32]	Finnish American Jewish	1 005	0, >13	25^※^	*								0.008^※※^
Present study	Chinese	356	0-96	15	4.3	6.1	7.8	9.4	10.8	27.6	40.6	38.0	0**

^*^ Not mentioned in the study; ^**^ The likelihood ratio test; ^※^ skewed to the extent of >70:30 or <30:70; ^※ ※^*F* test.

§allelic ratios: ≥ 4:1 or 80% predominant expression of one allele.

^☆^ The χ^2^ test; ^☆☆^ age constitution: neonates (*n* = 162); younger adults (25–32 y) (*n* = 67); older adults up to 60 y (*n* = 66).

The mechanism of SXCI is largely unknown. It is believed that a large proportion of SXCI cases result from the selection for, or against, alleles on the active X chromosome. Such selection may depend on the expression of the gene as well as its interactions with other genes [33]. SXCI may also occur when the size of the pool of the embryonic precursor cells undergoing X-chromosome inactivation is too small to avoid stochastic variation [33]. This can explain, at least partly, the existence of SXCI in some neonates. In addition, SXCI may be attributable to relatively small selective advantages such as X-chromosome rearrangements and mutations in X-linked genes [34,35].

When considering its pathogenic pathways, the skewing that is putatively associated with the unbalanced X-chromosome inactivation of precursor cells in early embryogenesis, is considered to be inborn SXCI, whereas the skewing that occurs during adulthood and is prevalent with age, is designated as acquired SXCI [5]. The former occurs at a low frequency (<5%) in normal females and is responsible for the neonate SXCI cases, although its significance is unknown. The latter, we believe, occurs due to the selection or structural alterations in X chromosomes.

Our data also suggests that SXCI occurs less frequently in the apparently healthy females in China (15%, with CR ≥ 3 as the moderate criteria) than in those in Western countries, as listed in Table 2. The reason for this difference is unknown. Ethnic genetic variation may be one explanation. Minor differences in some genomic structures including STR and single-nucleotide polymorphism (SNP) sites among different ethnic groups have been reported [36,37], which may modulate the process of SXCI occurrence in females. In the present study, 356 informative cases were examined, and the results showed that their ages, ranging from 2 days to 96 years, followed a Gaussian distribution. The SXCI frequency, as provided herein, can reflect the overall status in healthy Chinese females.

While SXCI occurrence was associated with the autoimmune diseases including autoimmune thyroid diseases [38-40], and systemic sclerosis [41], this phenomenon has been linked to development of a few of malignant solid tumours [33] in females. In 1999, Buller *et al.* reported that patients with invasive ovarian cancer had an increased frequency of SXCI compared to those without a detectable fully malignant ovarian tumor [18]. In a survey by Kristiansen *et al.*, SXCI (CR ≥ 10) frequency was shown to be markedly increased in young patients (≤45 years) with breast cancer (13%) compared to that of the control group (1%) [16]. The similar phenomenon was also observed in familial breast cancer patients without a detectable *BRCA-*1 or *BRCA-*2 mutation [17].

Additionally, it is well known that esophageal carcinoma (EC) is one of the most prevalent cancers in the world with characteristic regional variation. In the past 4 years, several genome-wide association studies (GWAS) in Chinese populations [42-44] and Japanese individuals [45] revealed a few of functional SNPs, which encode metabolic enzymes for carcinogens associated with enhanced EC risk. Recently, another GWAS and genome-wide gene-environment interaction analysis of EC was also carried out in 2,031 affected individuals (cases) and 2,044 controls with independent validation in 8,092 cases and 8,620 controls in the Chinese population [20]. Similar to the previous studies [42-45], the GWAS confirmed a few of new EC susceptibility loci and the genetic contribution to EC through interaction with enviroment [20], However, all the authors were obliged to convery that EC was such a complex disease, and the direct genetic contribution to EC risk factors and its relationto other factors should be discussed more deeply [42], and more studies are needed to explore the underlying mechanisms.

In the present study, SXCI (CR ≥ 3) frequency was determined to be as high as 35.7% in young patients (≤ 40 years) with esophageal cancer, being significantly higher than that in the corresponding control group (7.6%), though the size of subjects detected was relatively small. Moreover, the average age at diagnosis in the cancer patients with SXCI was 16 years younger than that in the patients without SXCI. In the previous study of our group, SXCI was also observed more frequently in the female patients with lung cancer below 50 years (CR ≥ 3, 35.7%; CR ≥ 10, 7.9%) compared to that in the reference group (CR ≥ 10, 7.6%; CR ≥ 10, 1.2%). The cancer patients with SXCI, whether defined as CR ≥ 3 or CR ≥ 10, were 10 years younger on average than those without SXCI. Based on the data, SXCI was considered to be a predisposing factor for the early onset of lung cancer [19]. For this reason, follow-up was conducted after the survey for all of the 265 apparently healthy women recruited into the reference group in March 2005. Among the 27 subjects whose CR values were determined to be ≥3, a 34-year-old female (CR = 9.0) was diagnosed as pulmonary squamous cell carcinoma in April 2006 and died in July 2006, with the histological type being squamous cell carcinoma and the clinical stage being stage IV, while no malignancy was detected for the others (unpublished data).

## Conclusion

In conclusion, our data prove that SXCI is a predisposing factor also for the early development of esophageal carcinoma. We consider that SXCI may be used as a useful parameter to assess susceptibility of females to several solid tumours, including the early development of esophageal carcinoma.

## Competing interests

The authors declare that they have no competing interests.

## Authors’ contributions

QS and GG designed the study. GL and TJ participated in the design and coordination, performed the molecular genetic evaluation, and drafted the manuscript. And WZ and LG performed the statistical analysis, and joined into drafting the manuscript. All the patients were followed up by YT and HL. GL, QS and GG all contributed to improving the draft of the manuscript. All authors have read and approved the final manuscript.
